# The Effect of Heat Treatment on the Tribological Properties and Room Temperature Corrosion Behavior of Fe–Cr–Al-Based OPH Alloy

**DOI:** 10.3390/ma13235465

**Published:** 2020-11-30

**Authors:** Omid Khalaj, Ehsan Saebnoori, Hana Jirková, Ondřej Chocholatý, Ludmila Kučerová, Jan Hajšman, Jiří Svoboda

**Affiliations:** 1 Regional Technological Institute, University of West Bohemia, Univerzitní 8, 301 00 Pilsen, Czech Republic; hstankov@rti.zcu.cz (H.J.); skal@rti.zcu.cz (L.K.); janh@rti.zcu.cz (J.H.); 2 Department of Materials and Metallurgy, University of West Bohemia, Univerzitní 8, 301 00 Pilsen, Czech Republic; saebnoor@kmm.zcu.cz (E.S.); chochola@kmm.zcu.cz (O.C.); 3 Institute of Physics of Materials, Academy of Sciences of the Czech Republic, Žižkova 22, 616 62 Brno, Czech Republic; svobj@ipm.cz

**Keywords:** oxide precipitation hardened (OPH), heat treatment, corrosion, wearing, tribology, Fe–Cr–Al

## Abstract

The microstructure, mechanical, tribological, and corrosion properties of Fe–Cr–Al–Y-based oxide-precipitation-hardened (OPH) alloy at room temperature are presented. Two OPH alloys with a composition of 0.72Fe–0.15Cr–0.06Al–0.03Mo–0.01Ta–0.02Y_2_O_3_ and 0.03Y_2_O_3_ (wt.%) were prepared by mechanical alloying with different milling times. After consolidation by hot rolling, the alloys presented a very fine microstructure with a grain size of approximately 180 nm. Such a structure is relatively brittle, and its mechanical properties are enhanced by heat treatment. Annealing was performed at three temperatures (1000 °C, 1100 °C, and 1200 °C), with a holding time from 1 to 20 h. Tensile testing, wear testing, and corrosion testing were performed to evaluate the effect of heat treatment on the behavior and microstructural properties. The grain size increased almost 10 times by heat treatment, which influenced the mechanical properties. The ultimate tensile strength increased up to 300% more compared to the initial state. On the other hand, heat treatment has a negative effect on corrosion and wear resistance.

## 1. Introduction

Oxide-dispersion-strengthened (ODS) or oxide-precipitation-hardened (OPH) alloys are extinguished by their significant high strength and advanced creep properties, as well as reasonable resistance to swelling, hardening, and embrittlement [1,2]. They consist of a metallic matrix formed of nickel alloys, iron aluminum alloys, and ferritic and austenitic steels with dispersed nanosized oxide particles. The new Fe–Cr–Al-based OPH steel has received significant attention because of its good corrosion resistance and high mechanical properties [3]. Aluminum and chromium are considered the most effective alloying elements for decreasing corrosion at high temperatures as well as at room temperature [4]. Forming a passive oxide layer can protect the surface against electrochemical corrosion and act as a barrier film.

Heat treatment can change the microstructure and phases in alloys. For OPH steel, heat treatment changes the matrix phase along with the precipitation. Heating can lead to grain growth and, sometimes, the formation of new phases. Lu et al. [5] studied the effect of heat treatment of Eurofer 97, Eurofer ODS, and T92 steels and showed that the grain size increases in T92 and Eurofer 97 steel with the increase of normalizing temperature, and the formation of Nb-rich and Ta-rich carbides and remains almost unchanged in Eurofer ODS steels. Ta enrichment in yttrium particles is also observed.

Previous research has mainly focused on ODS alloys and Fe–Cr-based materials processed via powder metallurgy [1,6,7,8,9]. Yttria is among the most important elements for providing strengthening and good thermal stability through oxides. However, La_2_O_3_, Ce_2_O_3_, ZrO_2_, and MgO are reported to provide good matrix strengthening [2,10]. Dual stabilization is also adopted in some cases to improve the mechanical properties of materials [9]. The volume fraction of dispersed particles in most cases is below 1%, and the mean particle size is in the range of 5–30 nm [2,11,12]. The outstanding high-temperature corrosion resistance of Fe–Al-based alloys is highly recommended for structural applications operating at high temperatures, offering comparably low costs, and several methods for their production and processing are widely available [13]. The fine-grained microstructure of ODS alloys was shown to be unstable at higher temperatures. Grain and particle coarsening under the appropriate heat treatment were reported by several authors [2,11,14,15,16,17,18,19,20,21,22,23,24]. The coarsening of the particles was shown to negatively affect material strength.

Grain coarsening was shown to be advantageous for the creep resistance of the alloy. The grain growth from 150–200 nm to 50 μm during an 8 h, 1000° heat treatment for Fe–Al–O ODS alloy is presented in [14]. In another study, the effects of heat treatments on dual-phase ODS steels’ properties consisting of ferrite and austenite formers, Cr, W, Ni, and C were investigated. It is reported that the oxide distributions and grain sizes of ODS steels can be significantly altered by heat treatment, which affect the strengths at elevated temperatures [25]. Al as a stabilizer of the ferritic phase, which improves the anticorrosion behavior by forming a protective Al_2_O_3_ layer on the surface and Cr, is considered the most effective in improving high-temperature oxidation resistance and room temperature corrosion [26].

Based on the literature review, it has been established that both corrosion resistance and mechanical properties are affected by grain structure, which is highly influenced by heat treatment. Thus, our study focuses on the corrosion resistance of Fe–Cr–Al OPH alloy. A series of corrosion and wear tests and tensile and hardness tests were performed on two variants of OPH alloys under different heat treatments, followed by metallographic analysis.

## 2. Experimental Procedure

### 2.1. Material Preparation

The new Fe–Cr–Al-based OPH steel was prepared by metal powders using powder metallurgy [19]. The difference compared to other ODS steels is the use of a higher content of yttrium nano-oxides and aluminum [27,28,29,30]. In the first step, powders of Fe, Al, Y_2_O_3,_ and other components (Table 1) are mechanically alloyed in a vacuum in a low-energy ball mill developed by the authors. The purity of the powder was 99.9%. Because of the vacuum in the mill, no oxygen was added during mechanical alloying, and the amount of oxygen was given by the input amount of yttrium. The mill has the possibility to be sealed, evacuated, or filled by gas. Mechanical alloying (MA) increases the density of defects in the powder’s matrix, leading to oxygen desolations in the matrix drastically by being trapped in the defects. In the next step, the MA powder was transferred to a low-alloy steel rolling container with a diameter of 20 mm, which had no contact to the air and was later evacuated then sealed by welding. As a next step, a hot rolling mill was used to process the prepared materials with three steps of hot rolling to a final OPH sheet approximately 2.5 mm thick, which was covered on both sides by a 0.3 mm thick scale from the rolling container. To investigate the influence of mechanical alloying time, the OPH steels were prepared with different milling times. As a result, the effect of the milling time on the homogeneity of the obtained material and the effect of contamination from the milling balls from the bearing steel on the mechanical properties could be observed.

### 2.2. Speciemen Preparation

After rolling, the container was removed from the OPH steel. Heat treatment (HT) was performed in an atmospheric furnace. Three heating temperatures, 1000, 1100, and 1200 °C, were chosen, with holding times of 0, 5, and 20 h. The sample preparation process was optimized to fulfill all the requirements and cover all types of tests. Square samples (20 mm × 20 mm) were prepared to be fit to the advanced corrosion cell. Circular samples with a diameter of 10 mm and a thickness of 2 mm were prepared for the wear test.

Standard microtensile pieces were cut to cover the OPH alloy variants’ mechanical and microstructural tests. The cross-section of the active part was 1.2 mm × 2 mm, with a length of 5 mm. All necessary samples were subsequently cut to desired forms by means of a water jet system. The samples were cut parallel to the rolling direction (in the longitudinal direction), and the thickness of the specimens was approximately 2 mm after grinding.

### 2.3. Testing Equipment and Procedure

A UHL/VMHT hardness tester (Walter Uhl, Asslar, Germany) and a servohydraulic MTS thermomechanical simulator (MTS, Eden Prairie, MN, USA) were used to perform the mechanical tests. The purpose-built convertors (UWB, Pilsen, Czech Republic) were manufactured by the authors to hold different sample sizes on the servohydraulic MTS. All the tensile tests were performed at room temperature (RT) with a constant strain rate of 0.001 s^−1^ to simulate static conditions. The hardness tests were performed with a load of 10 kg and a loading time of 11 s on the head of the polished samples’ surface. The average value was calculated from three measurements. The hardness was measured on the head of the pieces for the tensile test.

A scanning electron microscope (SEM), Zeiss Crossbeam 340-47-44 (Zeiss, Oberkochen, Germany), was used for the metallographic analysis of the samples. All the cross-sections were prepared using standard grinding and polishing processes. The final polishing step was performed with an oxide polishing suspension (OPS) to observe the very fine microstructure. Grain size measurement was made using the intersection method according to ASTM E112. After corrosion and wear resistance tests, the samples were analyzed by Zeiss Evo 25 (Zeiss, Oberkochen, Germany) and SEM Philips XL30 (Philips, Eindhoven, Netherlands), respectively.

A circular area with a diameter of 3 mm (A ≈ 7 mm^2^) was exposed to the electrolyte in a flat cell for the corrosion test. Before electrochemical tests, the surfaces were mechanically polished with up to 1200 grit SiC paper, and afterward, they were ultrasonically cleaned in acetone and washed with distilled water. The potentiodynamic anodic polarization tests were performed in a 3.5% NaCl aqueous solution at room temperature. An SP-150 BioLogic electrochemical instrument (BioLogic Science Instruments, Seyssinet-Pariset, France), a saturated calomel electrode (SCE), a counter electrode (Pt), and a working electrode were used for the electrochemical corrosion test. The potentiodynamic anodic polarization experiments were performed at a scan rate of 1 mV/min from 250 mV below the open-circuit potential (OCP) to 1000 mV above the OCP. All the samples were ultrasonically cleaned in acetone, washed with distilled ethanol, and observed with SEM and an optical microscope.

The steels’ tribological tests were performed using an Anton Paar TRB3 pin-on-disk tribometer (Anton Paar GmbH, Graz, Austria). The sample rotational speed was 300 rpm with a rotation radius of 3 mm. An alumina ball with a diameter of 6 mm and a load of 10 N was rubbed on the surface. During testing, the friction force was measured and recorded continuously. The friction coefficient was determined as the ratio of the friction force to the normal load. Before the tests, the surface of the samples was mechanically ground with 1200 grit SiC paper. After the tests, the samples were ultrasonically cleaned in ethanol for 5 min. The wear products were weighed using an analytical scale, and the grooves formed on the pieces were observed by SEM.

## 3. Results and Discussion

**Figure 1:**
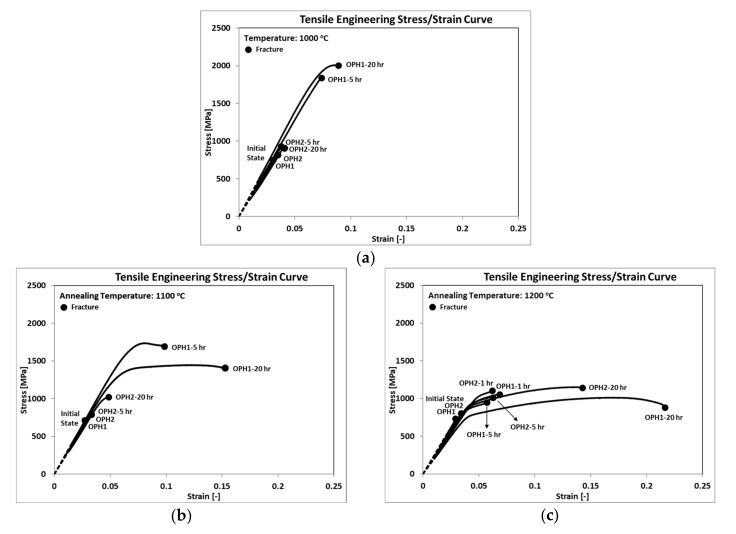
Flow curves after annealing at (**a**) 1000 °C; (**b**) 1100 °C; (**c**) 1200 °C.

After both variants’ heat treatment, the mechanical properties were investigated by performing the tensile test under a constant strain rate of 0.001 s^−1^. For each condition, three samples were prepared and tested, and the average values were reported as the tensile test result. Figure 1 shows the flow curves for both OPH1 and OPH2 under different HT, which are described in Table 1. It should be noted that all the tensile tests were performed at RT. Both UTS and elongation are significantly sensitive to heat treatment. For the UTS, the best result was achieved for OPH1 after 20 h of annealing at 1000 °C, whereas the best elongation was achieved for the same variant at 1200 °C after the same holding time.

Annealing at a higher temperature increased the ductility of OPH alloy, whereas at a lower temperature, a higher strength could be achieved regardless of milling time. Compared to the initial state (IS), the improvement in UTS varied from 20% after annealing at 1200 °C (Figure 1c) to more than 100% after annealing at 1000 °C (Figure 1a). The most effective annealing for OPH1 occurred at 1100 °C held for 20 h, which increased the UTS by almost 50%, whereas the elongation increased to 10%. The results also confirmed that longer milling time caused less improvement to the mechanical properties of OPH alloy, even after annealing at elevated temperatures. The UTS for OPH2 (longer milling time) after 20 h of annealing at 1000 °C and 1100 °C increased by almost 15% to 29%, respectively, compared to the initial state, whereas no improvement in elongation was observed. It seems that the improvement for OPH2 started at 1100 °C after 20 h of annealing (Figure 1b), which increased by increasing the annealing temperature to 1200 °C (Figure 1c). UTS increased by almost 30% and 43% after 5 and 20 h, respectively, compared to the initial state.

**Figure 2:**
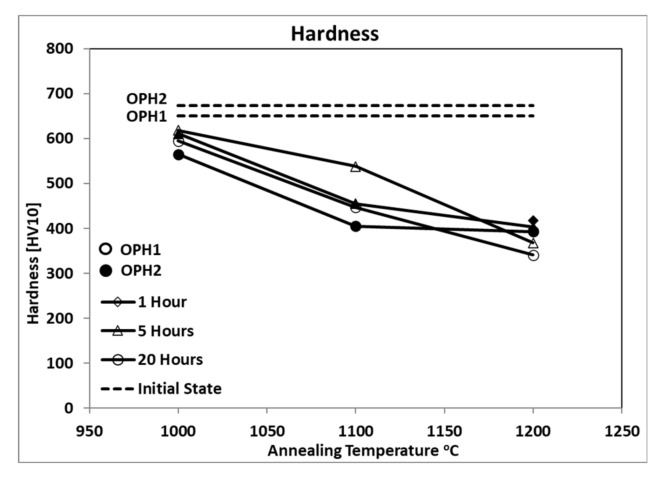
Hardness (HV10) at different conditions.

Figure 2 shows the hardness (HV10) results for both materials in the initial state and the samples after annealing with heat treatment at 1000 °C, 1100 °C, and 1200 °C, held at the times provided in Table 1. The hardness values of both variants demonstrate the ability of OPH alloys to resist plastic deformation at various elevated temperatures. As the ODS ferritic steels possess remarkable mechanical properties, such as high tensile and creep strength, OPH steels indeed have an extremely hard surface and high strength. The results reveal that annealing also affected the hardness of both variants of OPH alloys. Elevating the temperature to 1200 °C decreases the hardness by almost 30% compared to the initial state, whereas at 1000 °C, it decreases the maximum by 10%. OPH1, after 5 h annealing at 1100 °C, shows less reduction in HV10 than OPH2 under the same conditions. This could be attributed to the recrystallization process, which does not start in this situation for OPH1 [16].

The decrease in HV10 is more considerable in the first step of annealing from 1000 °C to 1100 °C. However, in the second step, from 1100 °C to 1200 °C, less reduction occurred. On the other hand, the long holding time has more influence on the hardness value of OPH alloys. Holding for 20 h up to 1100 °C decreases the hardness two times more than similar conditions with shorter holding times, whereas at 1200 °C, a longer holding time has the same influence as a shorter time.

**Figure 3:**
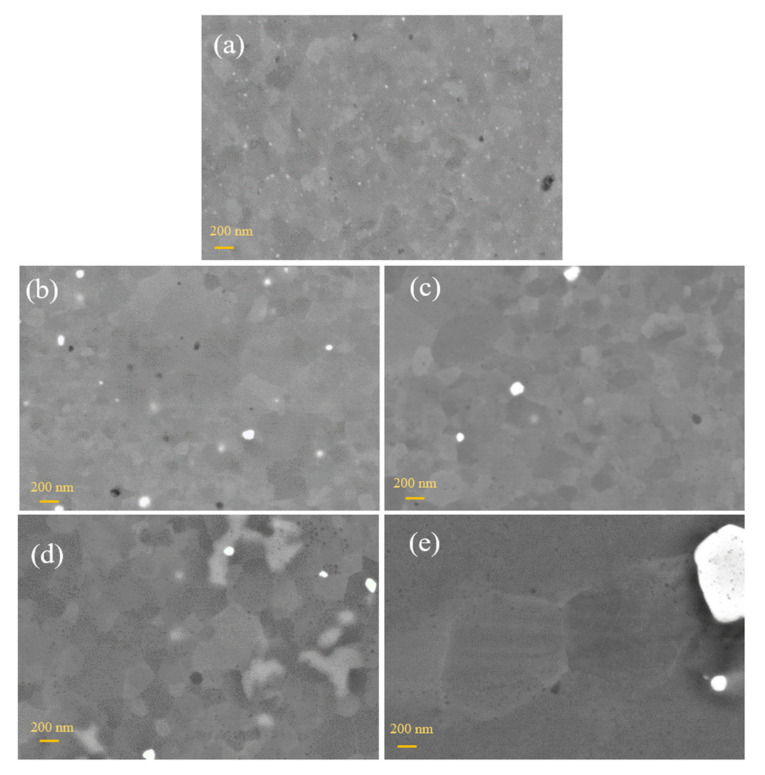
SEM images of OHP1 steel (**a**) as-rolled (**b**) and heat treated at 1000 °C/20 h, (**c**) 1100 °C/20 h, (**d**) 1200 °C/1 h, and (**e**) 1200 °C/20 h (for all images: BSD detector, EHT—10 kV, WD—approx. 4 mm).

Backscattering electron SEM micrographs (OPS polishing) for as-rolled and heat-treated samples at 1000 °C, 1100 °C, and 1200 °C are provided in Figure 3. All samples demonstrated a ferritic microstructure with distributed oxide precipitates.

**Figure 4:**
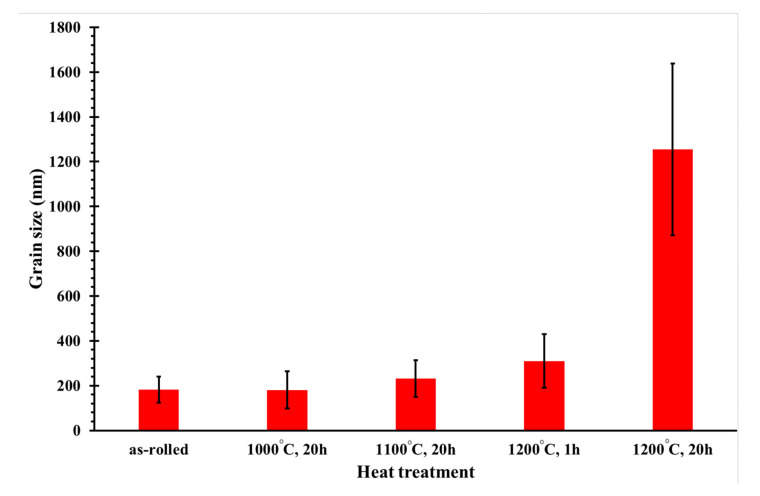
Dependence of mean grain size on the heat treatment conditions.

In the rolled state without heat treatment, the consolidated homogenous structure with equiaxed grains was observed without visible defects and pores (Figure 3a). The grains were very fine and approximately 180 nm. Very small nanoprecipitates were detected inside and on the boundaries of the grains. A previous study confirmed that these are yttrium nanoprecipitates [21]. The structure’s recrystallization was almost invisible at the lower annealing temperatures of 1000 °C and 1100 °C and prolonged the grain growth (Figure 3b,c). The grain size was increased from 182 nm in the as-rolled state to 232 nm after annealing at 1100 °C (Figure 4). With the higher annealing temperature, the precipitates were coarser (Figure 3b–e), and white particles were detected mainly at the grain boundaries. Complete recrystallization and significant grain growth were obtained at 1200 °C and a holding time of 20 h (Figure 4). The grain size increased up to 1254 nm.

**Figure 5:**
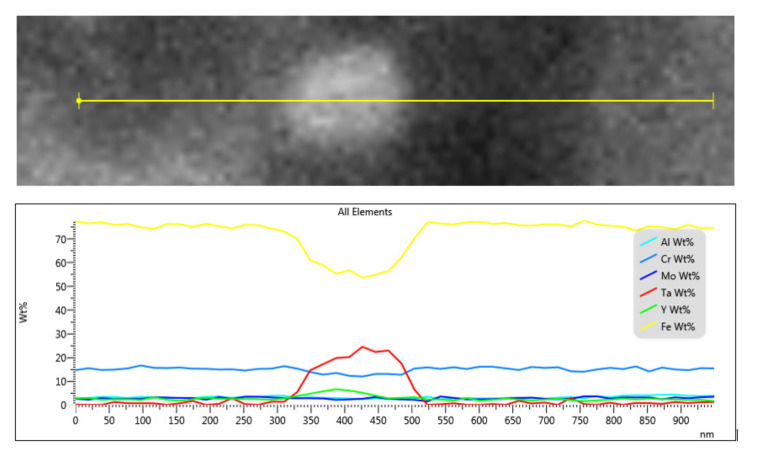
EDS line scan analysis of a precipitate in the microstructure of the OPH1 steel (as-rolled state, without heat treatment, EHT—10 kV, WD—6.4 mm).

The EDS (Energy Dispersive Spectroscopy) analysis results confirmed that the large white particles are tantalum-rich (Figure 5). For the EDS analysis, a detector from Oxford Instruments (Abingdon, UK) with AZtechSynergy evaluation software was used. After annealing at higher temperatures, dark particles were also observed more often in the structure. This was mainly due to their growth during heat treatment.

**Figure 6:**
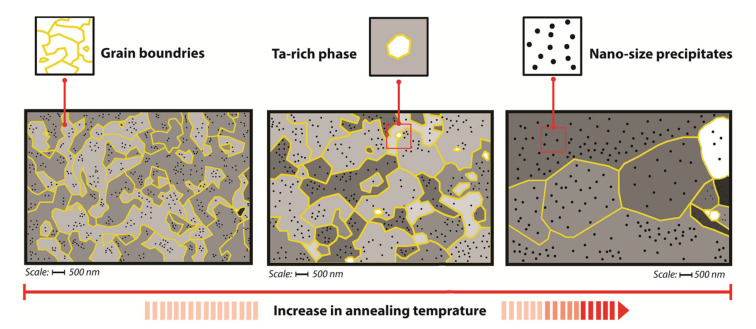
Schematic drawing of changes in microstructure occurring during annealing of the OPH steels.

The effect of the annealing temperature on the recrystallization of the structure is apparent in Figure 6. The increase in annealing temperature leads to gradual recrystallization of the microstructure. At higher annealing temperatures, complete recrystallization and significant grain coarsening occur. Precipitates also grow.

**Figure 7:**
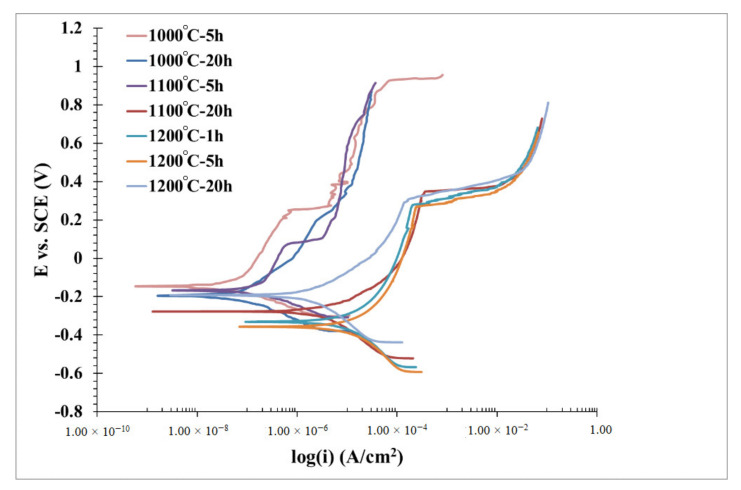
Potentiodynamic polarization curves for the OPH1 samples in a 3.5% NaCl aqueous solution with different heat treatment conditions.

**Figure 8:**
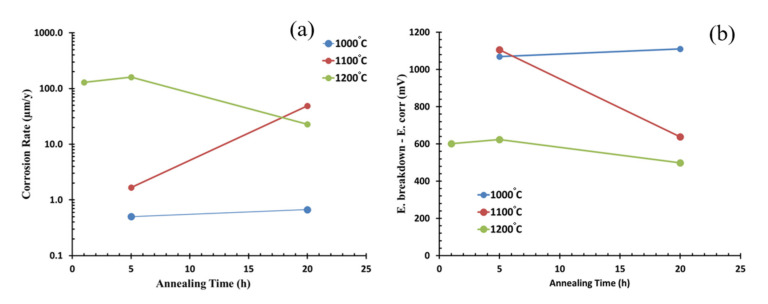
Corrosion rates (**a**) and the breakdown and corrosion potential differences (**b**) versus annealing temperature for the OPH1 samples plotted from data of Table 2.

Figure 7 shows the potentiodynamic polarization in a 3.5% NaCl solution of OPH1 alloy after different heat treatments. All the samples show passive corrosion followed by a breakdown and active corrosion in anodic branches. At a glance, it can be seen that the curves somehow shifted to the right by increasing the annealing time and temperatures. The extracted data from the Tafel calculation is presented in Table 2. As observed from the data, an increase in time and annealing temperature leads to a decrease in corrosion resistance. The values of corrosion rates and the breakdown and corrosion potential differences versus annealing temperature are plotted in Figure 8. The higher corrosion rate of the alloy annealed at higher temperatures is due to the formation of larger grain sizes responsible for the growth of oxide precipitates at the grain boundaries regions and grain growth (Figure 8a). Grain boundaries and precipitates are susceptible to the onset of corrosion and the formation of microgalvanic cells, exerting their influence on the corrosion current density and breakdown potential by prolonging the heat treatment time at higher temperatures. The corrosion rate increased by approximately 100 times as the heat treatment temperature increased from 1000 to 1200 °C. This effect has already been reported for many ferrous and nonferrous alloys [31] and can be attributed to grain growth and precipitation formation, resulting in microgalvanic cells’ formation. With developing grain boundaries, the onset of corrosion and weakening of the passive layer led to a significantly lower passive breakdown potential. In changing the temperature of heat treatment from 1000 to 1200 °C, this value reaches approximately 500 mV. Changes in corrosion rate are more severely affected by heat treatment temperature than by heat treatment time, but between the three heat treatment temperatures of 1000, 1100, and 1200 °C, the effect of time is more noticeable at medium temperatures. This can be attributed to the inadequate temperature of 1000 °C in terms of activation energy for microstructural changes. At a temperature of 1200 °C, the intensity of the changes is such that the grain growth occurs quickly, and then over time, up to 20 h, more changes are restricted. Similarly, it significantly shrinks the passive corrosion area in the OPH alloy (Figure 8b).

**Figure 9:**
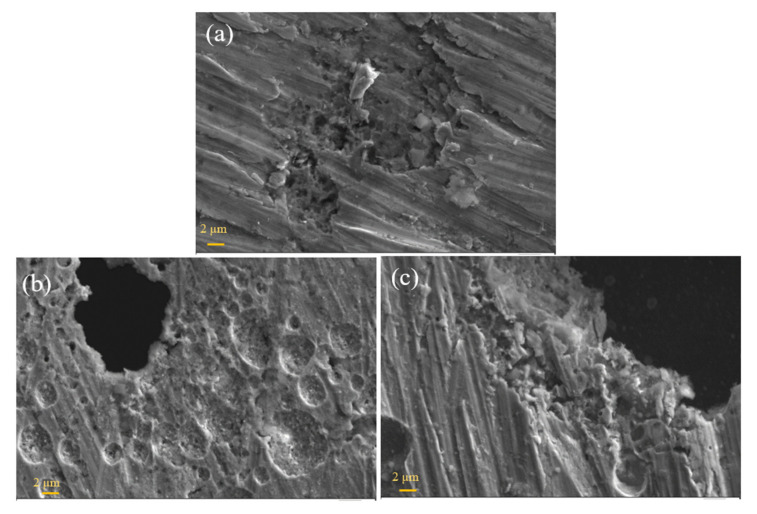
SEM images of the corroded surface for OPH1 heat treated for 20 h at 1000 °C (**a**), 1100 °C (**b**), and 1200 °C (**c**) after the corrosion test (for all images: SE detector, EHT—15 kV, WD—approx. 9 mm).

The surface morphologies of the OPH1 samples heat-treated for 20 h at 1000 °C, 1100 °C, and 1200 °C after the polarization tests are shown in Figure 9. Severe pitting corrosion and some localized corrosion can be observed with increasing annealing temperature. As reported [32], the Cr in the alloy could form a Cr_2_O_3_ passive oxide film and the formation of Al_2_O_3_ by the Al content, which could prevent the matrix from further corrosion. Moreover, the breaking-down of the passive film could also dominate the pitting behavior and impair the corrosion resistance [33,34]. It is also reported that galvanic corrosion around the grain boundaries and precipitates dominated the corrosion process. Annealing leads to the formation of bigger dispersoids and enhances microgalvanic corrosion [20].

**Figure 10:**
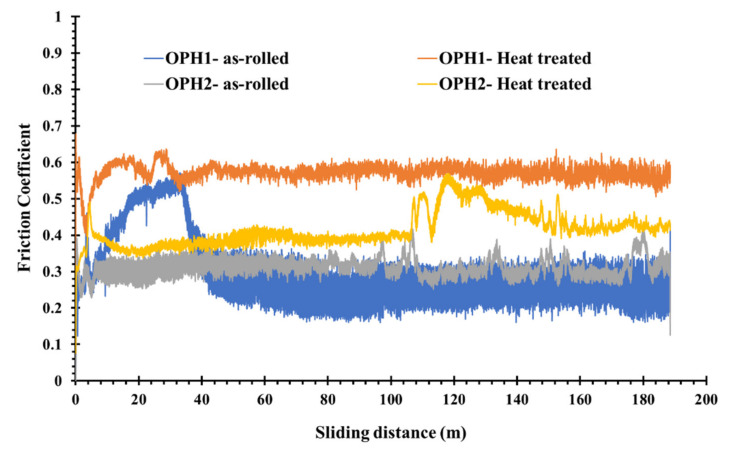
Friction coefficient vs. sliding distance during the pin-on-disk test.

Figure 10 shows the coefficient of friction (COF) for the as-rolled OPH1 and OPH2 samples heat-treated at 1200 °C for 20 h when sliding against the alumina ball in dry conditions. The ratio of the friction force determines the COF to the loading force on the ball. The results reveal that the COF increases with heat treatment. Higher friction leads to more increased wear and, consequently, less wear resistance. Between the two steels, OPH1 has a higher COF than OPH2 in the heat-treated form. This also corresponds to the results of the hardness measurements. Due to heat treatment, hardness decreased from approximately 600 HV10 for the as-rolled state at 450 HV10 after annealing at 1200 °C/20 h. The hardness itself is also affected by the milling time of the powders before rolling, which leads to higher homogeneity and better mechanical properties [35].

The analysis of COF curves leads to distinguishing three zones or regions of friction and wear (Figure 10). In the beginning, the COF increases sharply and decreases to a fluctuating value around a relatively constant rate. The first zone is the accommodation period, whereas the COF increases rapidly, and the roughness of the steel surface is reduced by plastic deformation [36]. A significant decrease in the COF defines the second period. The derbies generated by the steel surfaces’ frictional wear probably act as the third body and a solid lubricant. The third and final period corresponds to the COF stabilization, which fluctuates around a constant value. Some increase and decrease in the third period are attributed to the abrasive role of fragmented and oxidizes during the wear test.

All the wear behavior of the samples suggests that the abrasive wear mechanism is dominant during the test and some plastic deformation and adhesive mechanism. According to Archard’s law, the material’s volumetric loss is inversely proportional to its hardness value [37]: the higher the hardness of the material, the smaller the volume loss. For the studied alloys, because the heat treatment causes a decrease in hardness values, the sliding wear increases, according to Archard’s law.

The fluctuation in COF is closely related to the adhesive wear of the ODS samples. Firstly, the contact area grows with increasing lateral force over joint growth. The two sides remain in adhesive contact, causing a “stick.” When the applied force exceeds the adhesive strength, the joint breaks, and a rapid “slip” of the two surfaces occurs. This “slip–stick” cycle is repeated, accounting for the varied oscillation in the friction trace [38].

**Figure 11:**
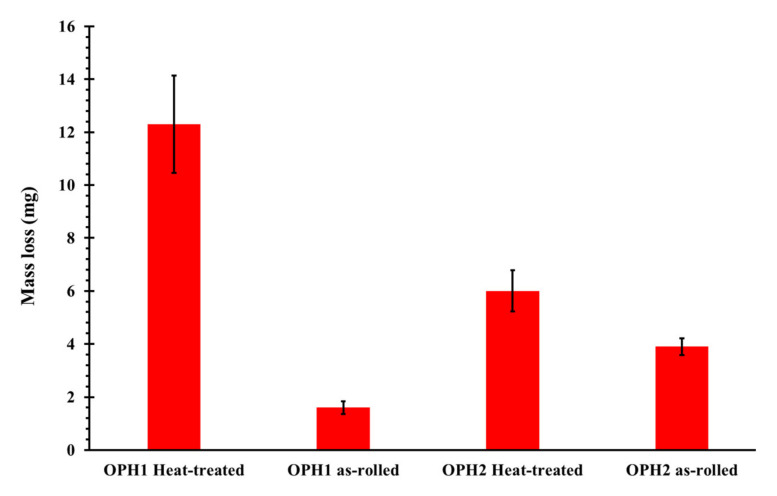
Mass loss measured from the pin-on-disk test.

The samples’ mass loss with a 10 N load and a sliding distance of 200 m is illustrated in Figure 11. As can be seen, the amount of mass loss is affected by heat treatment. The heat treatment at 1200 °C for 20 h significantly decreases hardness, which corresponds to our results (Figure 4). The heat-treated OPH2 sample shows more resistance to wear than the OPH1 sample with similar heat treatment. This can be attributed to a few differences in composition during the production process. During mechanical alloying, 5–10% of the material of the milling balls are ground from the balls and contaminate the powder [30]. As the C content in the ball bearings reaches approximately 1%, the mechanically alloyed powder is contaminated by 0.05–0.1% of C, which leads to the formation of Cr- and Mo-rich, stable carbides precipitating after static recrystallization at the grain boundaries. These carbides negatively influenced the mechanical properties [21]. However, the hardness increased, which also affects wear resistance.

**Figure 12:**
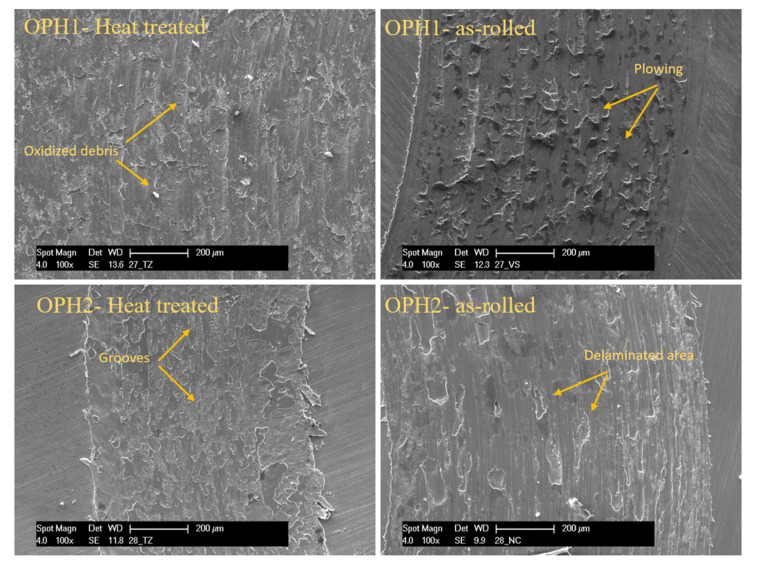
SEM images of wear tracks on the surface after the pin-on-disc test for OPH1 and OPH2 before and after heat treatment.

As shown in Figure 12, continuous sliding marks with ridges and plastically deformed grooves are visible on the worn surface. The dominant wear mechanism is abrasive wear with extension of plastic deformation or “plowing”. The plowing amount depends on the material’s strength, whereas the harder as-rolled samples show fewer plowing signs than the corresponding heat-treated samples. If the material has a higher hardness, the plowing impression will be mild, as shown in the figure. As-heat treated steels exhibit lower hardness values and hence undergo more plowing compared to as-rolled steels.

**Figure 13:**
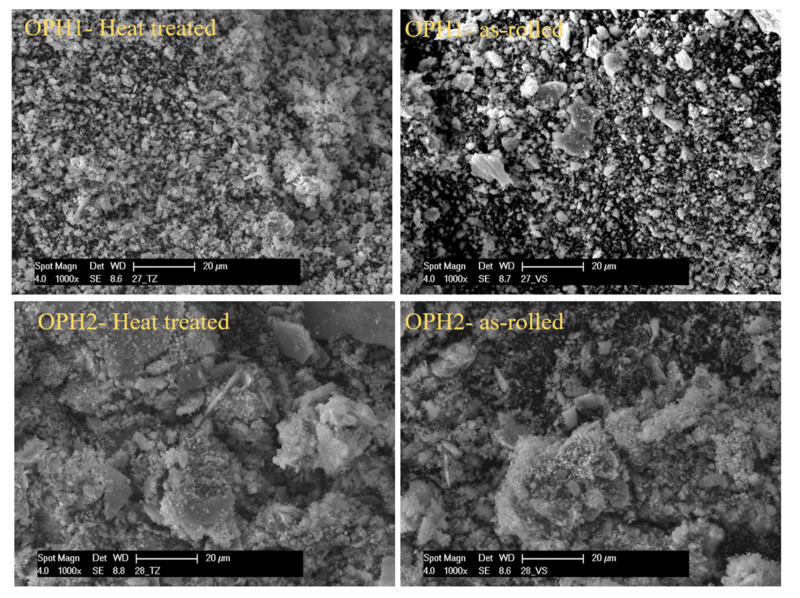
SEM micrographs of wear debris after abrasive wear (after pin-on-disc test) for OPH1 and OPH2 before and after heat treatment.

Figure 13 shows the SEM images of wear debris collected during the pin-on-disk test. Wear debris consists of very fine particles of a micrometer-order. The debris shapes are flakes, finely sized, and agglomerated, as seen in Figure 13. The flake-type debris was derived from delamination wear and more detected in as-rolled samples, which could be due to the higher hardness value of as-rolled samples. Without heat treatment, the material is also much more brittle and prone to brittle failure.

## 4. Conclusions

The effect of heat treatment on the mechanical properties, corrosion resistance, and tribological behavior of two oxide-precipitation-hardened (OPH) steels with Cr, Al, Mo, Ti, and Y was studied. The alloys were prepared by mechanical alloying and consolidated by hot rolling. Through this process, an ultra-fine-grained structure was obtained with a grain size of approximately 180 nm. To obtain a fully recrystallized structure with grains over 1000 nm, annealing at 1200 °C with a holding time of 20 h was necessary. The heat treatment caused a decrease in the hardness from 700 to 400 HV10. The change of the grain size and structure recrystallization had an impact on the mechanical properties. In the rolled state, a tensile strength of around 750 MPa was reached with almost no ductility. The highest tensile strength of 2000 MPa was obtained after annealing at 1000 °C with a holding time of 20 h for OPH1 steel. On the other hand, the highest ductility was observed for a fully recrystallized structure after annealing at 1200 °C/20 h.

Heat treatment had a negative influence on the corrosion and wear resistance. Electrochemical corrosion resistance decreased with an increase in heat treatment duration and temperature. This was caused by the increase of the grain size due to recrystallization after annealing.

## Figures and Tables

**Table 1:** Material properties (RT—room temperature).

Material No.	Milling Time (h)	Rolling Temp. (°C)	Annealing	Chemical Composition (wt.%)
OPH1	150	925	RT, 1000, 1100, and 1200 °C at 0, 5, and 20 h	0.72Fe–0.15Cr-0.06Al–0.03Mo–0.01Ta –0.02Y_2_O_3_
OPH2	230	925	RT, 1000, 1100, 1200 °C 0, 5, and 20 h	0.72Fe–0.15Cr-0.06Al–0.03Mo–0.01Ta–0.03Y_2_O_3_

**Table 2:** Electrochemical data extracted from the polarization curves (Figure 6).

Annealing Temperature/Time	E_corr_ vs. SCE (mV)	i_corr_ (µA/cm^2^)	β_a_ (mV/dec)	β_c_ (mV/dec)	C. Rate (µm/y)	E_br_ vs. SCE (mV)	E_br_ − E_corr_ (mV)
1000 °C/5 h	−155	0.043	238	77	0.5	914	1069
1000 °C/20 h	−191	0.057	152	104	0.7	920	1111
1100 °C/5 h	−178	0.143	403	87	1.7	929	1107
1100 °C/20 h	−290	4.186	163	199	49.0	349	639
1200 °C/1 h	−337	11.071	281	234	130. 0	265	602
1200 °C/5 h	−361	13.671	288	242	160.0	263	624
1200 °C/20 h	−186	1.971	165	206	23.0	312	498
